# Social Marketing to Enhance Community Empowerment and Ownership for a Successful Implementation of the “Big Catch-Up” in Togo in 2025: A Mixed-Methods Study

**DOI:** 10.3390/vaccines14050447

**Published:** 2026-05-18

**Authors:** Soliou Badarou, Aimé Serge Dali, Kokou Herbert Gounon, Lorraine Shamalla-Hannah, Amevegbe Kodjo Boko, Xavier Richard Sire, Erinna Corinne Dia

**Affiliations:** 1 United Nations Children’s Fund (UNICEF), Togo Country Office, Lome P.O. Box 80827, Togo; 2 Division of Health System and Policy, Institut National de Santé Publique, Abidjan BP V 47, Côte d’Ivoire; 3 Department of Global Health, University of Washington, Seattle, WA 98195, USA; 4 Department of Public Health, Faculty of Health Sciences, University of Lomé, Lome 1515, Togo; 5 African Center for Research in Epidemiology and Public Health, Lome 4089, Togo; 6 United Nations Children’s Fund (UNICEF), Afghanistan Country Office, Kabul P.O. Box 54, Afghanistan; 7 Division of Immunization, Ministry of Health, Public Hygiene, Universal Health Coverage and Insurance, Lome BP 386, Togo

**Keywords:** social marketing, zero-dose, under-vaccinated, vaccination coverage, community engagement, Big Catch-Up, codesign, Togo

## Abstract

**Introduction:** The COVID-19 pandemic disrupted immunization services in Togo, resulting in 69,672 “zero-dose” and 24,846 “under-vaccinated” children by the end of 2023. This study assessed the effectiveness, acceptability, and feasibility of a social marketing approach deployed during the 2025 Big Catch-Up initiative in Togo. **Methods:** A convergent mixed-methods study was conducted in 17 priority health districts. The quantitative component compared vaccination coverage before and after the intervention using administrative data. Chi-squared test for linear trend compared district-level coverages, and statistical significance was set at *p* < 0.05 for all tests. The qualitative component used in-depth interviews with key informants to collect data, followed by thematic content analysis. The intervention was grounded on the social marketing framework with 4 pillars (4Ps): Product, Price, Place, and Promotion. **Results:** Coverage increased dramatically: Penta1 from 1% to 64%, Penta3 from 1% to 45%, MR1 from 4% to 50%, and MR2 from 6% to 49% (all *p* < 0.001). Togo ranked 3rd out of 24 African countries for Penta1 progress. The approach demonstrated high community acceptability, with Vaccination Monitoring Committees praised as being culturally appropriate. Key concerns included sustainability and resource constraints. **Conclusions:** Social marketing was associated with increased community adherence and immunization coverage improvement. However, long-term sustainability requires the institutionalization of community structures with domestic funding and continued health system strengthening.

## 1. Introduction

The COVID-19 pandemic has severely disrupted routine immunization services worldwide, creating an unprecedented immunity gap. Between 2019 and 2021, approximately 67 million children missed some or all of their routine vaccinations, marking the largest decline in childhood immunization in three decades globally [1]. This decline has led to an alarming increase in the number of “zero-dose” children, from 12.9 million in 2019 to 18.1 million in 2021 [2] and 14.3 million in 2024 [3]. In the WHO African Region, vaccination coverage with the third dose of diphtheria–pertussis–tetanus vaccine (DPT3) has fallen from 74% in 2019 to 72% in 2021, jeopardizing decades of progress [4]. This regression has created pockets of vulnerability conducive to outbreaks of measles, polio, diphtheria, or yellow fever [5,6].

Sub-Saharan Africa bears the heaviest burden of vaccine-preventable child mortality and accounts for the majority of “zero-dose” children worldwide [7]. Disruptions related to COVID-19 have exacerbated this already precarious situation. In response, WHO, UNICEF, and Gavi, the Vaccine Alliance, launched the global “Big Catch-Up” (BCU) initiative in April 2023 as an essential immunization recovery plan with three objectives: reach children missed between 2019 and 2022 (partially due to the pandemic) with required antigens, restore vaccination coverage to at least the 2019 levels, and strengthen vaccination systems to achieve the Immunization Agenda 2030 goals (IA2030) [8]. Adopted at the World Health Assembly in 2020, IA2030 sets ambitious targets: to achieve 90% vaccination coverage at the national level and 80% in each district by 2030, with a particular focus on reaching “zero-dose” and “under-vaccinated” children [9].

In Togo, the Vaccination Coverage Survey (VCS) conducted in 2024 revealed vaccination coverage rates of 90.3% for DPT3, 80.8% for the first dose of the combined Measles/Rubella vaccine (MR1), and only 57.7% for the second dose of the combined Measles/Rubella vaccine (MR2). Although these rates showed an improvement over previous years, disaggregated analysis revealed significant geographical disparities, with some disadvantaged regions having coverage below 70% for Penta3 [10]. The external review of the EPI conducted in 2019 had already highlighted several structural weaknesses requiring action: shortcomings in the monitoring and tracking of “zero-dose” and “under-vaccinated” children, low community involvement in routine immunization, persistent vaccine hesitancy and rumors, and insufficient knowledge among mothers about the diseases targeted by the EPI, the immunization schedule, and the importance of completing the full course of immunization [11]. These shortcomings, exacerbated by disruptions related to the COVID-19 crisis, led to 69,672 “zero-dose” and 24,846 “under-vaccinated” children in Togo, reflecting the global pattern where approximately 67 million children missed some or all of their routine vaccinations during the pandemic [1].

Faced with this critical situation and regional disparities in vaccination coverage, Togo joined global efforts to catch up, restore, and strengthen pre-COVID-19 vaccination coverage levels by implementing the “Big Catch-Up” (BCU) initiative in 2025.

Social marketing has been increasingly applied to public health challenges, including vaccination programs [12,13]. Evidence on the effectiveness of social marketing in immunization programs is growing. In China, Zheng et al. applied a social marketing strategy to COVID-19 vaccination and reported a reduction in vaccine hesitancy from 52% to 3.1%, with coverage reaching 94.8% [12]. In South Korea, Hong et al. documented 94.8% second-dose and 71.3% third-dose COVID-19 vaccination coverage following a structured social marketing campaign [14].

Despite the critical importance of the BCU initiative in addressing the post-COVID-19 immunization gap in Africa, the scientific literature documenting social marketing approaches deployed in this specific context remains extremely limited. This case study on the Togolese experience in this regard has several justifications: it will provide evidence on the effectiveness of social marketing approaches in improving immunization demand and services in a West African context; help guide future immunization demand programming in Togo and inform national immunization policies [15]; and also serve as a reference for other African countries facing similar post-COVID-19 vaccination challenges, facilitating adaptation and replication [16]. Finally, it will help support resource mobilization efforts to sustain and scale up the approach.

We hypothesize that social marketing can significantly contribute to improving vaccination coverage among “zero-dose” and “under-vaccinated” children. This study aims to evaluate the effectiveness of a social marketing approach in increasing vaccination coverage among “zero-dose” and “under-vaccinated” children in priority districts in Togo in 2025, and to assess the implementation process by exploring acceptability and feasibility among various stakeholders.

## 2. Methods

### 2.1. Design and Period

A mixed convergent study combining quantitative and qualitative approaches was used. The quantitative component compared immunization indicators before and after implementation of the accelerated social marketing strategy. The qualitative component used key-informant in-depth interviews (IDI) to explore the acceptability and feasibility of the strategy. The choice of a mixed convergent design was deliberate and grounded in implementation science theory. While quantitative indicators capture measurable proxies of acceptability and feasibility at scale, they cannot explain the mechanisms through which the intervention was accepted or rejected, nor identify the contextual conditions that enabled or constrained implementation. Acceptability and feasibility are multi-dimensional theoretical constructs whose assessment requires access to stakeholder attitudes, perceived cultural fit, and subjective experiences, dimensions that are not reducible to numerical indicators. Findings from both strands were integrated at the interpretation stage. The study covered two distinct periods: the baseline period (January–June 2025), which is the initial phase of the BCU implementation in Togo, and the intervention period (August–December 2025) that followed the mid-term review and strategic reorientation in July. Data collection occurred in January 2026.

### 2.2. Conceptual Framework

This study was guided by the RE-AIM (Reach, Effectiveness, Adoption, Implementation, Maintenance) framework, a comprehensive model for planning and evaluating public health interventions. Developed by Glasgow et al., the RE-AIM framework provides a systematic approach to assess multiple dimensions of intervention success and translation into real-world settings. The framework assessed intervention reach among target populations, effectiveness on vaccination outcomes, adoption by actors, implementation fidelity, and potential for long-term sustainability [17].

### 2.3. Study Setting

**Figure 1:**
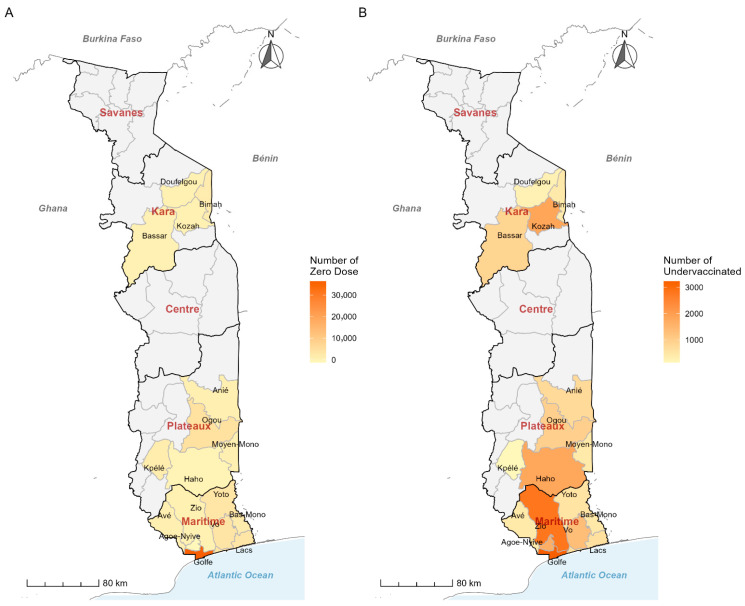
Target regions and districts for “zero-dose” (**A**) and “under-vaccinated” (**B**) children for the Big Catch-Up in Togo in 2025.

The intervention was implemented across four priority health regions of Togo: Grand Lomé, Maritime, Plateaux, and Kara, all of which accounted for more than 70% of the country’s zero-dose and under-vaccinated children. The two health districts of The Grand Lomé region, Agoè-Nyivé and Golfe, were included, whereas 6 priority health districts (Zio, Vo, Yoto, Bas-Mono, Lacs, Avé) were selected in the Maritime region). Figure 1 presents the baseline characteristics of the 4 priority regions selected for intensified intervention. The majority of zero-dose children (*n* = 36,394) were located in the Golfe district (Figure 1A), and the majority of under-vaccinated children were located in the Golfe (*n* = 3230) and Zio (*n* = 2799) districts (Figure 1B). In total, the intervention targeted 17 districts with 157 health catchment areas identified as the highest priority based on the concentration of zero-dose and under-vaccinated children.

### 2.4. Intervention: Social Marketing Approach

The “social marketing” approach has demonstrated effectiveness in addressing various public health challenges, including immunization, with evidence showing significant improvements in vaccine coverage when properly implemented [12,13].

**Figure 2:**
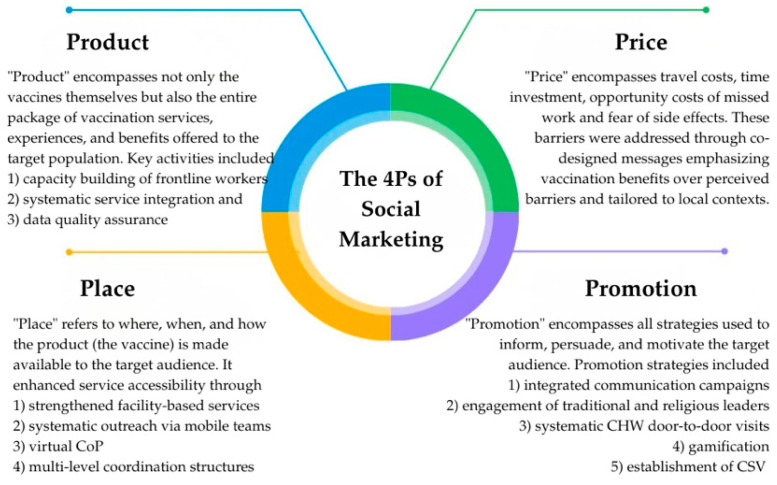
The 4Ps of social marketing, adapted from Glasgow et al. [17]. CoP = community of practice, CSV = community-based vaccination monitoring committees.

In Togo, a comprehensive social marketing strategy was designed and deployed from August to December 2025 following a mid-term review of the BCU that identified key bottlenecks during the initial phase (March–July 2025): persistent vaccine hesitancy, a weak zero-dose and under-vaccinated children tracking system at the community level, poor logistics and immunization data quality limited coordination and accountability at all levels, and a lack of integration of the BCU activities with other routine health services (malnutrition screening, antenatal care, etc.). This strategy integrated the four pillars of the marketing mix, also known as the 4Ps (Product, Price, Place, and Promotion), to synergistically address service-side, supply-side, and demand-side barriers to immunization (Figure 2). This integrated, multi-dimensional approach distinguishes social marketing from isolated health promotion campaigns or service delivery improvements. By simultaneously addressing what is offered (Product), barriers to adoption (Price), accessibility (Place), and community engagement/demand (Promotion), the intervention created mutually reinforcing pathways to behavior change. Data quality was ensured through monthly supervisory visits at the health facility level, triangulation of BCU-specific reporting forms against DHIS2 entries, and data review meetings at the district level. To mitigate double-counting across quarters, children were tracked by unique household identifiers and cross-checked against facility vaccination registers.

### 2.5. Quantitative Component

**Study Population and Eligibility Criteria:** “Zero-dose” children (*n* = 69,672) and “under-vaccinated” children (*n* = 24,846) in priority districts constituted the study population. These were children aged 0–59 months at the time of the intervention (March–December 2025), residing in Togo, and classified as “zero-dose” or “under-vaccinated” based on a review of vaccination records. When an official vaccination record was unavailable, verbal recall from the caregiver was used to ascertain vaccination history. All “zero-dose” and “under-vaccinated” children identified through active case finding during the BCU implementation period were included. They were identified by CHWs during household visits; each CHW maintained a dedicated tracking register listing identified children by name, age, and vaccination status, updated during each home visit. The entire target population of 69,672 “zero-dose” and 24,846 “under-vaccinated” children was the denominator for coverage calculation.

**Data Collection and Analysis:** Quantitative data were collected from multiple sources: the DHIS2 (District Health Information Software 2), the health facility registers, the Big Catch-Up specific reporting forms, and a digital dashboard jointly developed by WHO and UNICEF regional offices in Africa for closely monitoring the Big Catch-Up initiative in all eligible countries. DHIS2 is Togo’s national health information system, capturing monthly aggregated routine vaccination statistics from all health facilities by antigen, age group, and health facility. Health facility registers are vaccination registers at the facility level that were reviewed for data completeness and accuracy. Big Catch-Up specific reporting forms are custom-designed BCU forms to capture intervention-specific process indicators not systematically available in DHIS2. The digital dashboard is a real-time platform to monitor vaccination progress, vaccine stock levels, and cold chain functionality. Comparative regional BCU data and aggregated data on BCU performance (Penta1, Penta3 coverage by quarter) for all 23 African countries implementing the initiative were extracted from this platform (Microsoft Power BI) [18].

Data were analyzed using R version 4.5.0 [19]. Categorical variables were described in terms of frequency and proportion. Vaccination coverage was estimated as a percentage. Chi-squared test for linear trend (Cochran-Armitage test) [20,21,22] compared district-level coverage across quarters before and during the accelerated intervention period for each antigen (Penta1, Penta3, MR1, MR2). Togo’s quarterly BCU progress was compared with that of other African countries using descriptive statistics and ranking. Statistical significance was set at *p* < 0.05 for all tests.

**Operational Definitions:** *“Zero-dose” children* are defined as those who have not received even a single vaccine shot. For operational purposes, Gavi defines zero-dose children as infants who have not received the first dose of diphtheria, tetanus, and pertussis-containing vaccine (DTP1) by the end of their first year of life [23]. *“Under-vaccinated” children* are defined as infants who have not received the third dose of DTP-containing vaccine (DTP3) by the end of their first year of life [23]. *Vaccination coverage* is defined as the proportion of a given population that has been vaccinated in a given time period. It is estimated for each vaccine, and for multi-dose vaccines, for each dose received (e.g., DTP1, DTP2, DTP3). It is usually presented as a percentage [24]. *Priority health areas* are defined as catchment areas of health facilities with the highest burden of “zero-dose” and “under-vaccinated” children within each priority district.

### 2.6. Qualitative Component

**Inclusion Criteria, Diversification Criteria, and Sampling:** Participants were eligible for qualitative interviews if (1) they were directly involved in planning, implementation, supervision, or had benefited from the social marketing intervention; and (2) were willing and able to provide informed consent. Purposive sampling was used to maximize heterogeneity across roles and geographic locations. Participants were identified through consultation with the country’s Immunization Division and UNICEF’s program officers, who provided lists of key informants at various levels. The selection was deliberately designed to capture perspectives across levels of the health system. Based on qualitative research guidelines [25,26] and resource constraints, the target sample size was set to 10 key informants from 5 health districts (Agoè-Nyivé, Bas-mono, Golfe, Vo, and Yotto) distributed across 2 regions (Grand Lome and Maritime) The reasons behind this purposive sampling are as follows: 8 (47%) out of the 17 priority health districts are in the Grand Lomé and Maritime regions, whereas 77 (49%) out of 157 catchment areas are in the Grand Lomé and Maritime regions. Plus, the Grand Lomé, which is the capital city, and the Maritime region account for 57% of the overall population targeted by the intervention across the 4 regions. This trend is similar for priority health districts where key informants were selected.

**Data Collection and Analysis:** Qualitative data were collected using multiple complementary methods. *In-depth interviews (IDI)*: semi-structured interviews lasting on average 35 min were conducted with key informants. Interview guides were developed and covered topics such as the BCU implementation process, the four intervention dimensions, facilitators and barriers encountered, coping mechanisms, perceived effectiveness, acceptability and feasibility, and recommendations. Interviews were conducted in French or local languages, audio-recorded with consent, and supplemented with detailed field notes. *Informal interviews*: conducted with program officers from UNICEF and the Ministry of Health. *Document review*: program documents were systematically reviewed, including BCU strategic plans, training materials, communication materials, and supervision reports. This provided contextual information and supported data triangulation. Interviews occurred in private locations convenient for the participants (offices, health facilities, and remotely). Participants received no financial compensation but were reimbursed for transport costs when applicable.

Qualitative data were analyzed using thematic content analysis following a systematic process: *Transcription and translation*: all audio recordings were transcribed verbatim within 48 h of collection. Local language transcripts were translated into French by bilingual translators with public health familiarity and reviewed for accuracy against audio recordings. *Coding*: the analysis was conducted using a hybrid coding approach, combining deductive coding based on the conceptual framework with inductive coding to capture emergent themes from the data. A codebook was developed, and line-by-line coding was performed using RQDA software (version 0.5) [27], with codes systematically applied to relevant text segments. Related codes were grouped into categories and broader themes. *Interpretation*: themes were interpreted in light of the conceptual framework and existing literature. Mechanisms linking intervention components to outcomes were explicated. Findings were reported using thick descriptions with illustrative verbatim quotes.

### 2.7. Ethical and Regulatory Considerations

This case study received ethical approval from the Institutional Review Board of the School of Health Sciences at the University of Lomé in Togo [No. 409/2026/CE-FSS/19/01]. All participants signed a consent form. The confidentiality of the data collected was respected. To ensure anonymity, no information that could identify the participants was used.

## 3. Results

### 3.1. Quantitative Results

#### 3.1.1. Implementation Characteristics

The implementation was assessed through the monitoring of key process indicators (Table 1).

**Vaccination Monitoring Committees (CSVs) established and functional:** The intervention enabled 100% of the target to be achieved. A total of 157 CSVs were successfully established across the 157 priority health catchment areas, meeting the objective of one CSV per priority facility. Each CSV comprised 15 members representing diverse community segments, including neighborhood chiefs, CHW, health facility staff, and community leaders. Of the 157 CSVs established, 100% were classified as functional based on criteria including: holding at least 80% of the planned meetings during the intervention period, documented participation in the codesign of vaccination service organization, and active involvement in identifying and referring zero-dose children. A CSV meeting at least two of these three criteria is considered functional. Functionality status was assessed by district-level supervisors. No independent external verification was conducted.

**Community feedback mechanisms:** the CSV served as the primary platform for community feedback and accountability. Across the 157 CSVs, a total of 487 community feedback items (from suggestions to complaints) were recorded during the intervention period. These were related to vaccination schedule inconveniences (38%), vaccine stockouts or cold chain issues (25%), provider attitudes (18%), long waiting times (12%), and other issues (7%). Of these, 421 (86.4%) were addressed and resolved during subsequent CSV meetings. Unresolved complaints (13.6%) were primarily related to structural issues requiring district or regional-level decisions. Resolution was defined as the documentation of a specific response or corrective action in the CSV meeting within two subsequent meeting cycles. Resolution status was assessed and documented by the CSV secretary during meetings. No independent external verification was conducted.

**Capacity building of vaccinators:** A total of 1917 actors were trained, including 223 vaccinators (11.6%), and 1570 CHWs and community relays (81.9%). All participants received training in at least two evidence-based communication techniques. Religious leaders additionally received a briefing on the intervention principles. Table 1 presents the distribution of trained actors by category.

#### 3.1.2. Evolution of Vaccination Coverage

**Temporal Distribution of Vaccination Coverage**

**Figure 3:**
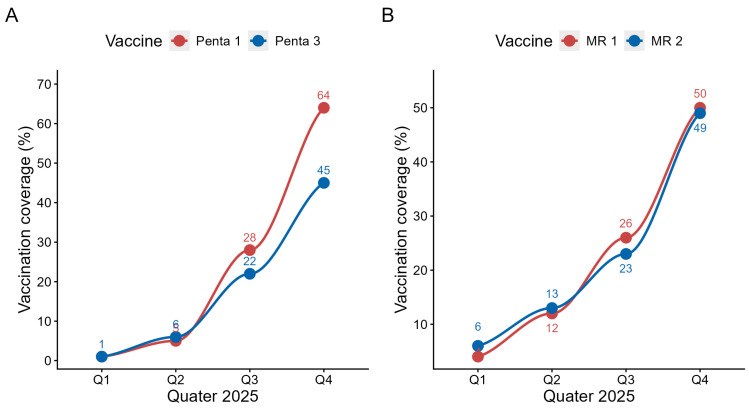
Temporal distribution of vaccination coverage of Penta (**A**) and MR (**B**) in Togo in 2025. The curve is used as a visual guide rather than an exact representation of intermediate values.

The accelerated social marketing intervention resulted in dramatic improvements in vaccination coverage for all tracer antigens. Figure 3A presents the evolution of Penta1 and Penta3, and Figure 3B presents the evolution of MR1 and MR2 coverage from January to December 2025.

Penta1 coverage increased progressively from 1% in Q1 (pre-acceleration phase) to 64% by Q4. The August–December phase alone contributed 59 percentage points of the 63% annual progress, representing 94% of the total annual gain achieved in just 5 months. The analysis revealed a statistically significant inflection in the trend (*p* < 0.001). For Penta3 coverage, a similar acceleration pattern was observed. Coverage rose from 1% (Q1) to 45% (Q4). The acceleration phase contributed 40 out of 44 percentage points, representing 91% of the total annual gain. The inflection in the trend was statistically significant (*p* < 0.001).

MR1 coverage also showed dramatic gains, increasing from 4% (Q1) to 50% (Q4). The August–December period accounted for 38 out of 46 percentage points gained, representing 83% of the total annual gain. The pre- and post-intervention trend increase was statistically significant (*p* < 0.001). MR2 demonstrated substantial improvement from 6% (Q1) to 49% (Q4). The intervention period contributed 36 out of the 43 percentage points gained, representing 84% of the total annual gain. The increase in the trend was statistically significant (*p* < 0.001).

**Spatial Distribution of Vaccination Coverage by Health District**

**Figure 4:**
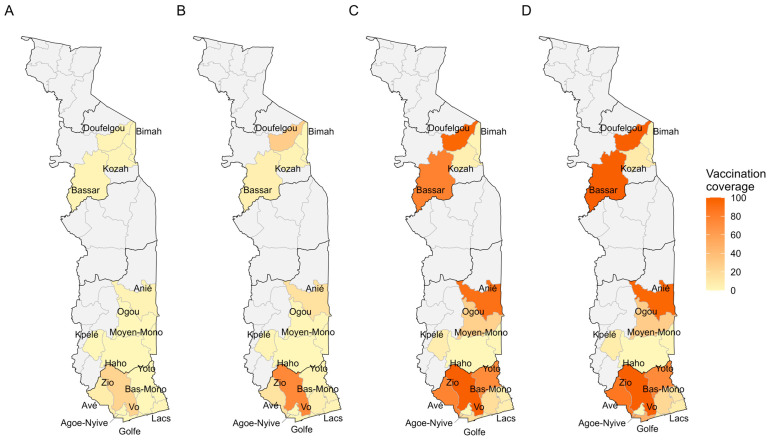
Spatial distribution of vaccination coverage of Penta1 by quarter in Togo in 2025. (**A**) Penta 1, Q1; (**B**) Penta 1, Q2; (**C**) Penta 1, Q3; (**D**) Penta 1, Q4.

**Figure 5:**
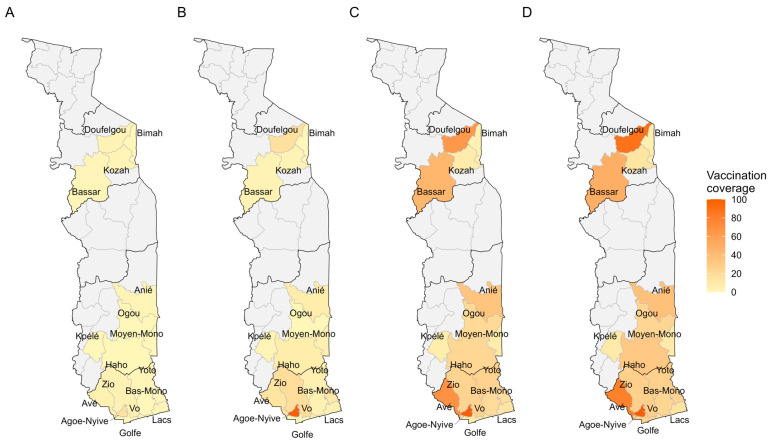
Spatial distribution of vaccination coverage of Penta3 by quarter in Togo in 2025. (**A**) Penta 3, Q1; (**B**) Penta 3, Q2; (**C**) Penta 3, Q3; (**D**) Penta 3, Q4.

Figure 4 shows progress in vaccination coverage in Penta1 and Figure 5 in Penta3 by district and quarter. For Penta1, there was a clear increase in vaccination coverage between Q2 (Figure 4B) and Q3 (Figure 4C), with the best performance in the health districts of Zio, Bassar, and Doufelgou (100%) in Q4 (Figure 4D). For Penta3, there was also a clear increase in vaccination coverage between Q2 (Figure 5B) and Q3 (Figure 5C), with the best performance in the health districts of Agoè-Nyive (100%), Doufelgou (94%), and Avé (82%) (Figure 5D).

#### 3.1.3. Comparison of Togo’s Performance with Other African Countries

**Figure 6:**
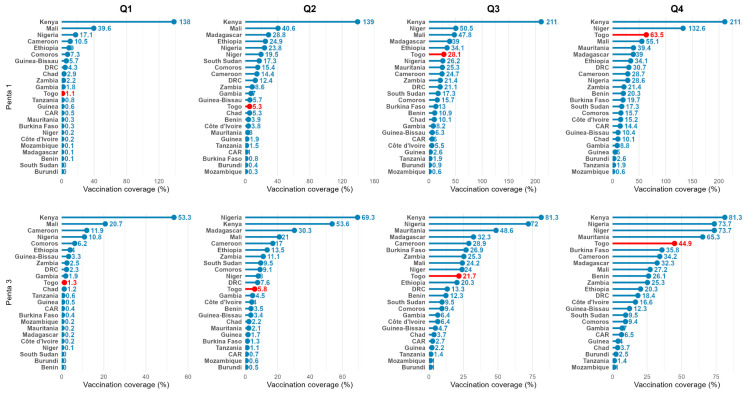
Comparative performance of African Big Catch-Up countries for Penta1 and Penta3 coverage in 2025.

Togo’s performance in the BCU initiative was benchmarked against 24 other African countries implementing the program. Figure 6 presents the comparative regional performance for Penta1 and Penta3 coverage progression.

For the Penta1 progression (January–December 2025), Togo ranked 3rd out of 24 African BCU countries, with 63.5% coverage in December 2025. Notably, Togo dramatically improved from 12th place in Q1 to 3rd place in Q4. Togo’s half-yearly gain of 58.2 percentage points between Q2 and Q4 was the second-highest acceleration among all countries, exceeded only by Niger (113.1 points).

For Penta3 progression (January–December 2025), Togo ranked 5th with 44.9% coverage, placing it in the middle tier of performers. Kenya led dramatically with 81.3%, followed by Niger and Nigeria (both 73.7%). Togo’s Penta3 ranking lagged behind its Penta1 ranking (number 5 vs. number 3). However, strong acceleration was observed during Q2–Q4 with a jump from 5.8% (Q2) to 44.9% (Q4), placing the country among the top 5 performing countries.

### 3.2. Qualitative Results

#### 3.2.1. Sociodemographic Characteristics of Respondents

Of the 10 individual interview participants, 2 were female and 8 were male. Age ranged from 35 to 54 years; the median age was 44 years (IIQ = 40.75–49.75) and the median number of years of experience as a health professional was 11 (IIQ = 7–16). Professional roles included members of CSVs (*n* = 2), health promotion focal point (HPFP) (*n* = 2), health facility heads (HFH) (*n* = 1), immunization focal point (IFP) (*n* = 2), prefectural health director (PHD) (*n* = 1), member of COGES (management committees) (*n* = 1), and beneficiary (*n* = 1). Geographic diversity was achieved with participants from 5 health districts: Agoè-Nyivé, Bas-mono, Golfe, Vo and Yotto. Thematic saturation was assessed iteratively during data collection: after the 8th interview, no new major themes emerged, and the 9th and 10th interviews confirmed saturation across the predefined conceptual dimensions.

Table S1 provides an extensive overview of recurring themes and examples of quotes regarding the intervention’s acceptability, feasibility, barriers, and facilitators.

#### 3.2.2. Acceptability

The intervention demonstrated high levels of community acceptability across regions, with stakeholders at all levels expressing positive perceptions of the approach. The most recurring themes were: CSV as a culturally appropriate mechanism, Engagement of religious/traditional leaders, Gamification and recognition, and Behavioral change.

**CSV as culturally appropriate engagement mechanism:** The establishment of CSVs was praised as a culturally appropriate mechanism for community engagement that respected local decision-making structures while introducing systematic accountability. A health promotion focal point in Bas mono explained:

*“The community appreciated the CSV because they feel that their loved ones and parents are raising their awareness about something they expected to have side effects from. They see that it is the community that is reaching out to them to tell them that it is good to get vaccinated.”*

(IDI04-IFP-Golfe)

**Religious and community leader engagement appreciated:** The involvement of religious and community leaders was widely accepted and effective. A health promotion focal point in Bas mono district shared:

*“Some pastors asked children to bring their vaccination records to church. They checked them and found that a certain number of children in their communities had not been vaccinated. They see that there is a CHW in the area and entrust the children to them. As for the Vodou priests, it is in the convents and gatherings for ceremonies. For Imams, it is at the mosques. In each area, the Imams were invited. For example, in Attitongon, they passed on the information to the mosque. Some Imams brought children to the health facilities. They found, convinced (…) and accompanied the children.”*

(IDI02-HPFP-Bas-mono)

**Gamification and recognition ceremonies acceptable:** The performance-based recognition system and public ceremonies generated mixed reactions. High-performing districts and facilities appreciated the visibility and motivation. A health promotion focal point stated:

*“Because we won a trophy with a medal. We came to present it at the district level. At first, all the leaders were happy. Everyone on the CSV committees was happy with the activities they had carried out. When they saw the trophy, the village chiefs were very happy.”*

(IDI02-HPFP-Bas-mono)

However, low-performing districts expressed some concern about public discomfort. Nevertheless, overall, acceptability was high when recognition emphasized learning rather than only competition. A Prefectural Health Director confided:

*“When it comes to social marketing, sharing results should not be seen as something that will upset anyone. No, we are all here together, sharing our experiences. It is a place where those who are less successful can learn from the experiences of those who have done good work in the field to improve coverage.”*

(IDI05-PHD-Vo)

**Behavioral change:** Many behavioral changes were reported during and after the activities, with special mention of adherence to the vaccination schedule and a decrease in vaccine hesitancy. A member of CSV stated:

*“Hesitancy and other issues have changed. This has allowed us to have a larger number. Above all, adherence to schedules has also been important.”*

(IDI01-CSV-Agoè-nyivé)

#### 3.2.3. Operational Feasibility

The intervention was generally perceived as operationally feasible, though implementation challenges required adaptive solutions. The most recurring themes were: Simultaneous approach of awareness raising, research, and vaccination; Cascade training and home visits.

**Simultaneous approach of awareness raising, research, and vaccination:** In this context, it became apparent that awareness-raising efforts were not limited to a single task but were part of a continuum of integrated activities aimed at maximizing the impact of vaccination campaigns within the communities visited. A member of the COGES explained:

*“During vaccination, if we arrive in a region, we vaccinate them there, in the community. We set up a place under the trees or at the notable’s or chief’s house, and vaccinate them there. For some, we direct them to the Peripheral Health Unit. But there are certain areas that are a little far from the Peripheral Health Unit, so whenever we raise awareness, we vaccinate them there.”*

(IDI06-COGES-Yoto)

**Training implementation feasible with cascading approach:** Training 1917 actors within the compressed timeframe was achieved through a cascading model: master trainers trained focal points, who in turn trained district teams, who eventually trained facility-level actors and CHWs. A health facility head described:

*“… we were also trained at the beginning. There were ASC training sessions that followed. (…) we also trained them before sending them out into the field. For the community, we had an initial training session for all community members. (…) some members were trained as CSVs.”*

(IDI03-HFH-Golfe)

**Home visits operationally demanding but desirable:** Conducting such home visits over the project implementation period required significant CHW effort. This logistical and human aspect of home visits has been described as particularly demanding, but also perceived as essential. A beneficiary reported:

*“For my part, I was informed by a relative who is a community health worker. (…) He came to our house with two of his colleagues, who asked us if our children had been vaccinated. They explained the benefits of vaccination to us.”*

(IDI07-Beneficiary-Yoto)

#### 3.2.4. Perceived Barriers to Implementation

Despite overall success, several barriers hindered optimal implementation. The most recurring themes were: Geographical and infrastructural barriers, Organizational and resource barriers, Community and cultural barriers, and Sustainability concerns.

**Sustainability concerns:** Although some CSV members continued to do their work despite the end of the project, participants nevertheless raised substantial concerns about the sustainability of activities, pointing out that despite the positive effects observed in the short-term, CSVs may not be sustainable without the continued commitment of local authorities. This has led to active dialogue with mayors to explore mechanisms for long-term institutional and financial support. A Prefectural Health Director stated:

*“At present, we have all seen the impact on our activities. So, as of today, what we have done most is to approach local authorities, especially mayors, to see how they can help us to keep our activities going. (…) We are in discussions, and the grievances are on the mayor’s table.”*

(IDI05-PHD-Vo)

**Geographical and infrastructural barriers:** Hard-to-reach areas and displaced people faced significant access challenges as well as rainy weather conditions. Geographical barriers were consistently mentioned by actors as major logistical challenges. A Prefectural Health Director added:

*“The main difficulty was that the activity was carried out in rainy weather. And there were difficulties, especially in reaching certain areas.”*

(IDI05-PHD-Vo)

**Organizational and resource barriers:** Some participants expressed organizational and human resource concerns, particularly regarding the insufficient number of community workers available to carry out a sufficient number of visits, which could limit the number of children actually vaccinated in certain areas. A health promotion focal point worried:

*“But we also noticed that there weren’t enough CHWs assigned. (…) There were almost 30 CHWs with community relays. Only 15 were selected. So that bothered us a little. But we tried to manage it that way. And now we can see the number of vaccination team visits. (…) Because in some districts, this has somewhat hindered the number of children vaccinated. Because we have given six visits. And if the CHWs stick to these six visits, there will certainly be children who remain unvaccinated. We need to increase (…) the number of visits by vaccinators.”*

(IDI02-HPFP-Bas-mono)

#### 3.2.5. Perceived Facilitators

Multiple factors are perceived to have facilitated successful implementation:

**Awareness raising:** Awareness-raising activities helped to change the beneficiaries’ perceptions of vaccination, not only by providing clear information about its benefits, but also by tailoring the message to families’ living conditions and reducing individual hesitancy through personalized discussions. A beneficiary noted:

*“As my two children were not vaccinated, they offered us the vaccination. (…). They still managed to convince me of the benefits of vaccination and how important it is for our children’s lives. (…) When it comes to the message about vaccination, when you go to the hospital, the message gets across. However, when you are at home, and people come to visit, they explain things to you in a different way. And even when you are afraid, the fear dissipates.”*

(IDI07-Beneficiary-Yoto)

**Co-create approach and community engagement:** Unlike top-down campaigns, the co-creation methodology genuinely engaged communities in solution design, generating ownership, and increasing sustainability. A health facility head contrasted:

*“(…) We held meetings with the community. They were the ones who came up with key ideas for mobilizing the population around vaccination. (…) It is an advantage to involve people who live in the community (…) they understand that their health is in their own hands.”*

(IDI03-HFH-Golfe)

**Strong commitment from Government:** Close collaboration between the UNICEF technical team and the government leadership at the operational level created synergy. An immunization focal point noted:

*“UNICEF, which spearheaded the social marketing initiative, collaborated with the government. (…) The collaboration consisted of (…) pooling the various services (…) by inviting other sectors that UNICEF considered influential for the success of social marketing, notably the health promotion division and the immunization division of Ministry of Health (…). This was crucial.”*

(IDI04-IFP-Golfe)

### 3.3. Integration of Quantitative and Qualitative Results

Triangulation of quantitative performance data with qualitative actor experiences revealed convergent and complementary findings.

**Convergence on community engagement effectiveness:** Quantitative data showed 100% of CSVs were functional, and 86.4% of community feedback was addressed. Qualitative data explained *why* these mechanisms worked (cultural appropriateness of co-creation, respect for local decision-making) and *how* they translated into outcomes (generating ownership, enabling context-specific solutions, creating accountability).

**Complementarity on capacity building impact:** Quantitative results documented 1917 actors trained. Qualitative findings added depth, revealing that training such a large number of actors required a cascade training strategy. Furthermore, qualitative data revealed that the training’s impact extended beyond individual skill acquisition to creating a community where vaccinators, CHWs, and leaders mutually reinforced vaccination messages.

**Sustainability insights from divergent perspectives:** Quantitative data showed high CSV functionality during intervention (100%). Qualitative data revealed sustainability concerns: anticipated decline when external support ends, and the need for continued modest financial support.

The integrated mixed-methods design thus provided a comprehensive, nuanced understanding unattainable through either method alone: quantitative evidence of effectiveness, qualitative insights into mechanisms, acceptability, feasibility, contextual factors, and sustainability considerations.

## 4. Discussion

The quantitative results revealed substantial progress: Penta1 vaccination coverage rose from 1% in Q1 to 63.5% in Q4, and Penta3 coverage rose from 1% in Q1 to 44.9% in Q4, with a marked acceleration during the intensive intervention phase. Similarly, MR1 progressed from 4% to 50%, and MR2 from 6% to 49%. Regionally, Togo ranked 3rd out of 24 African countries for Penta1 progress and 5th for Penta3. Qualitative results showed strong community acceptance of the approach, particularly thanks to the CSV, which served as a culturally appropriate mechanism for community engagement. The involvement of religious and traditional leaders, combined with home visits and the simultaneous awareness-raising, research, and vaccination approach led to notable behavioral changes including reduced vaccine hesitancy and better adherence to the vaccination schedule.

We reported coverage increasing from 1% to 64% for Penta1 and from 1% to 45% for Penta3 in six months. Similar progress was reported in Angola, where an approach combining community mobilization and the involvement of traditional leaders increased DPT1 coverage from 75.8% to 90.2% and DPT3 coverage from 67% to 84% in one year [28]. This similarity in the progression of vaccination coverage suggests that intensified social mobilization strategies produce comparable effects in different African contexts. Rapid progress during the acceleration phase was also reported in Ethiopia, where the relaunch of the BCU in February 2025 resulted in the vaccination of nearly 100,000 children in just two months, increasing coverage from 16% to 24% of the target population [29]. These rapid accelerations demonstrate that well-coordinated strategic intensification can produce substantial results in a relatively short period of time.

We reported a high acceptability of CSV as a mechanism for community engagement. Similar results have been reported in Nigeria, where the Community Health Influencers, Promoters, and Services (CHIPS) program adopted a similar approach to community mobilization with convincing results: more than 4 million eligible children vaccinated between March 2021 and January 2023, including more than 700,000 who received Penta3 [30]. This could be explained by the community ownership of health interventions, a fundamental principle for reaching marginalized populations.

The involvement of religious groups, women’s groups, and traditional leaders proved crucial to our approach. A systematic review of immunization demand strategies in Africa reported similar results, showing that community mobilization using indigenous communication channels (town criers, religious leaders) significantly increases vaccination coverage [31]. This could be explained by the influence of these leaders as reliable sources of information, which helps to counter misinformation and build trust in vaccination services, particularly in rural areas where their authority is predominant.

The simultaneous approach of awareness-raising, screening, and vaccination was an important operational innovation that maximized efficiency. Carcelen et al. reported similar optimization approaches in a scoping review, notably the PIRI (Periodic Intensification of Routine Immunization) approach in India, which was organized for seven consecutive days each month [32]. By avoiding the delay between identification and vaccination, these approaches reduce dropouts and improve completion rates.

The use of the 4Ps of social marketing (Product, Price, Place, Promotion) in our vaccination context generated substantial increases of 1% to 45% for Penta3. Zheng et al., in China in 2023, used the same social marketing strategy for COVID-19 vaccination and reported a significant reduction in vaccine hesitancy from 52% to 3.1% and an increase in vaccination coverage to 94.8% [12]. Similarly, Hong et al., in South Korea in 2023, used the 4Ps strategy of social marketing and reported 94.8% coverage for the second dose and 71.3% for the third dose of COVID-19 vaccination [14]. These international results validate the applicability of this strategy to different contexts and populations.

The programmatic implications are manifold. For Togo, it is essential to capitalize on established CSVs and institutionalize them in the routine health system. Achieving genuine institutionalization of the CSV model will require addressing several interconnected dimensions. Regarding domestic financing for long-term sustainability, the costs must be integrated into district and municipal health budgets. Community incentives must be reconsidered. Gamification, understood as a new approach to managing a team, inspiring motivation in others, and fostering a positive work environment may offer a more sustainable model. Supervision capacity at the district level must be strengthened, and digital reporting tools could reduce the work burden. Health system capacity, particularly cold chain maintenance and vaccine stock management at the peripheral level, is a prerequisite for translating community demand into actual vaccination. The codesign and community engagement approaches should be extended to other health interventions to maximize investment in these structures. Strengthening the health information system, particularly the quality of immunization data, must continue to ensure accurate monitoring and data-driven planning. For other African countries facing similar challenges, this Togolese experience offers transferable lessons.

This study makes several contributions to the social marketing literature, particularly in the context of global health and immunization programs in low- and middle-income countries. We provide one of the first empirical demonstrations of the synergistic application of all four pillars of the social marketing mix within a national immunization catch-up program in sub-Saharan Africa. This study extends the application of the RE-AIM framework as a theoretical lens for evaluating social marketing interventions. By applying RE-AIM to assess a routine immunization program operating under severe resource constraints in West Africa, we demonstrate the framework’s adaptability and utility for implementation evaluation in this class of interventions. Our findings contribute to the emerging literature on the co-design as a social marketing tool. The Vaccination Monitoring Committees (CSVs) in this study functioned not merely as delivery channels, but as active co-designers of the intervention. From a methodological standpoint, this study demonstrates the added value of mixed-methods designs in social marketing evaluation.

### Strengths and Limitations

This evaluation has several methodological and conceptual strengths. The use of a mixed design combining quantitative and qualitative analyses provided a holistic understanding of the intervention. This methodological triangulation strengthens the validity of the conclusions. The evaluation was based on the integrated RE-AIM conceptual framework (Reach, Effectiveness, Adoption, Implementation, Maintenance). This structured theoretical approach enabled us to evaluate not only the effectiveness of the intervention, but also its reach, adoption by providers, fidelity of implementation, and potential for sustainability. This multidimensional evaluation goes beyond simple measures of vaccination coverage. The inclusion of 17 priority health districts in four regions representing more than 70% of the country’s “zero-dose” and “under-vaccinated” children ensures substantial geographical representativeness. This targeted approach, based on the burden of disease, optimizes resource allocation while generating data that can be generalized to areas with a high prevalence of unvaccinated children. The comparative analysis with 24 other African countries implementing the BCU provides an essential regional context for assessing Togo’s performance. This regional perspective allows Togo’s progress to be situated on a performance gradient and guides cross-country learning for continuous improvement.

However, certain methodological limitations must be considered. The absence of a control group limits the strict causal attribution to our intervention. The design adopted cannot completely exclude the influence of confounding factors such as competing interventions that may have contributed to the improvements observed. The short follow-up period does not allow for an assessment of the long-term sustainability of coverage gains or the sustainability of CSVs. The quality of administrative data is a significant limitation. Despite efforts to triangulate and improve data quality, inconsistencies may persist in some health facilities.

## 5. Conclusions

The social marketing approach deployed in Togo as part of the Big Catch-Up was associated with rapid and substantial increases in vaccination coverage among zero-dose and under-vaccinated children, contributing to the reduction in the post-pandemic immunization gap. For these gains to translate into sustainable progress toward the goals of the Immunization Agenda 2030, political and financial commitment from the government, the institutionalization of community mechanisms, and continuous strengthening of primary health systems are imperative. The Togolese experience confirms that vaccine equity in Africa is achievable, but requires political will, substantial domestic investment, and participatory approaches that place communities at the heart of vaccination strategies.

## Figures and Tables

**Table 1:** Actors involved in the Big Catch-Up and social marketing approach by category and region in Togo in 2025.

Region	Actors Involved ^1^			
	Vaccinators (*n* = 223)	CHW (*n* = 1570)	CSV Members (*n* = 157)	Overall (*N* = 1917)
Grand Lomé	66 (29.6)	330 (21.0)	33 (21.0)	429 (22.4)
Maritime	44 (19.7)	440 (28.0)	44 (28.0)	528 (27.5)
Plateaux	47 (21.1)	470 (29.9)	47 (29.9)	564 (29.4)
Kara	66 (29.6)	330 (21.0)	33 (21.0)	396 (20.7)

^1^ *n* (%).

## Data Availability

One part of the original data presented in the study is openly available in the Big Catch-Up Report Dashboard developed by the immunization regional working group (Microsoft Power BI) at https://app.powerbi.com/view?r=eyJrIjoiMWE2M2M3M2UtZDFhOC00MzE5LThmNjQtNDQwNmY4ZGY2NGEzIiwidCI6ImY2MTBjMGI3LWJkMjQtNGIzOS04MTBiLTNkYzI4MGFmYjU5MCIsImMiOjh9, accessed on 17 February 2026. The other part of data presented in this study are available on request from the corresponding author due to restricted access to Ministry of Health staff only.

## Supplementary Materials

The following supporting information can be downloaded at: https://www.mdpi.com/article/10.3390/vaccines14050447/s1, Table S1. Selection of verbatim quotes illustrating the identified themes.

## Abbreviations

The following abbreviations are used in this manuscript:

BCU	Big Catch-Up
CAR	Central African Republic
CHW	Community Health Worker
CoP	Community of Practice
COGES	Management Committee
CSV	French acronym for Vaccination Monitoring Committee
DHIS2	District Health Information Software 2
DTP1/DTP3	Diphtheria–Tetanus–Pertussis 1/3 (first/third dose)
DRC	Democratic Republic of the Congo
VCS	Vaccination Coverage Survey
EPI	Expanded Program on Immunization
GAVI	Global Alliance for Vaccines and Immunization, The Vaccine Alliance
HFH	Health Facility Head
HPFP	Health Promotion Focal Point
IA2030	Immunization Agenda 2030
IDI	In-Depth Interviews
IFP	Immunization Focal Point
MR1/MR2	Measles-Rubella 1/2 (first/second dose)
Penta1/Penta3	Pentavalent vaccine 1/3 (first/third dose)
PHD	Prefectural Health Director
PIRI	Periodic Intensification of Routine Immunization
RE-AIM	Reach, Effectiveness, Adoption, Implementation, Maintenance
